# How readable are Australian paediatric oral health education materials?

**DOI:** 10.1186/1472-6831-14-111

**Published:** 2014-09-02

**Authors:** Amit Arora, Andy SF Lam, Zahra Karami, Loc Giang Do, Mark Fort Harris

**Affiliations:** 1 Centre for Primary Health Care and Equity, Faculty of Medicine, UNSW Australia, Room 345, Level 3, AGSM Building, Gate 11, Botany Street, Randwick NSW 2052, Australia; 2 Faculty of Dentistry, University of Sydney, Westmead, NSW, Australia; 3 Sydney and Sydney South West Local Health District, Sydney, NSW, Australia; 4 Australian Research Centre for Population Oral Health, University of Adelaide, Adelaide, SA, Australia

**Keywords:** Dental caries, Oral health, Health literacy, Health communication, Leaflets

## Abstract

**Background:**

The objective of this study was to analyse the readability of paediatric oral health education leaflets available in Australia.

**Methods:**

Forty paediatric oral health education materials were analysed for general readability according to the following parameters: Thoroughness; Textual framework; Terminology; and Readability (Flesch-Kincaid grade level (FKGL), Gunning Fog index (Fog) and Simplified Measure of Gobbledygook (SMOG)).

**Results:**

Leaflets produced by the industry were among the hardest to read with an average readability at the 8th grade (8.4 ± 0.1). The readability of leaflets produced by the commercial sector was at the 7th grade (7.1 ± 1.7) and the government at the 6th grade (6.3 ± 1.9). The FKGL consistently yielded readabilities 2 grades below the Fog and SMOG indexes. In the content analyses, 14 essential paediatric oral health topics were noted and Early Childhood Caries (ECC) was identified as the most commonly used jargon term.

**Conclusion:**

Paediatric oral health education materials are readily available, yet their quality and readability vary widely and may be difficult to read for disadvantaged populations in Australia. A redesign of these leaflets while taking literacy into consideration is suggested.

## Background

Dental caries in children is an international public health problem [1]. Despite improvements in oral health over the last 20 years, dental caries is identified as one of the most prevalent chronic diseases of childhood especially for those from a disadvantaged background [2-4]. The most recent Child Dental Health Survey of Australia in 2007 reported that 46 percent of the 6-year-olds had one or more decayed, missing or filled primary tooth and 10 percent of those examined were found to have 10 primary teeth affected [5]. Data from the United Kingdom (UK) and the United States of America (USA) show a similar picture [6,7]. If left untreated, childhood caries can lead to reduced growth, nutritional and sleep problems, problems with eating, speaking, and learning, as well as the potential to disrupt family life [1].

A history of childhood caries is the most reliable predictor of future caries development which presents a large financial burden for the local health services and individual families [8,9]. It is therefore essential to prevent childhood caries before the subsequent need of resource intensive clinical interventions and treatments.

Parents are often the child’s first teachers and play a significant role in maintaining their child’s overall health by transferring health-related habits to their children. One possible solution to promote healthy habits in children is to motivate parents during the child’s early years of life as habits developed during the primary socialisation process are likely to be carried forward into adulthood [10-12]. However, developing good dental habits during early childhood is a complex process and is largely dependent on a broad range of individual, family and community level factors [13].

The dental professional team, government health departments and industry partners play a role in educating parents to support developing good dental habits. In order for parents to implement preventive oral health routine for their children, they must have adequate functional oral health literacy i.e. a person’s ability to read and understand written oral health education materials [14]. Although there is evidence that patients generally prefer written information [15,16], it is often noted that leaflets are poorly designed [17,18]. For example, it was noted elsewhere that the reading skills of parents of paedatric patients were several grades lower than their reported highest level of education [19]. As a result some authors [14,20,21] have suggested that the value of health education literature may be compromised by an individuals’ literacy skills and that this may hinder his/her ability to obtain, understand and act upon the key health messages.

In 2006, the Adult Literacy and Life Skills Survey in Australia documented that 59 percent of the population aged between 15 and 74 years scored below a level of literacy regarded as optimal for health maintenance [22]. The Australian Bureau of Statistics (ABS) Report also noted that people with lower levels of health literacy often belong to one or more of the following categories: lower socioeconomic class, lower income and/or education, migrants from non-English-speaking countries and living farther from metropolitan cities [22]. It has also been concluded elsewhere that lower levels of health literacy is associated with higher use of expensive care, emergency services and increased rate of hospitalizations [23]. Further, a recent systematic review reviewed the impact of parental health literacy on child health outcomes and concluded that lower levels of parental literacy were associated with poor child health outcomes [24].

Despite the current research on general health literacy, very few studies have examined oral health literacy. Jackson introduced the scope of the problem of lower level of parental oral health literacy and child oral health outcomes and suggested several methods of improving communication between the dental professional team and the parents [25]. Studies conducted in the UK and the USA reported that public dental education materials were difficult to read for their respective population [26-28]. However, to date none have investigated the readability of paediatric dental education materials in Australia. Therefore, the aim of this study was to examine the content and general readability of paediatric oral health education materials available in Australia.

## Methods

We contacted Australian State and Territory Health Department’s; industry partners; and commercial organisations for all possible oral health education leaflets (n = 40) pertinent to paediatric oral health. Two were produced by the Australian Dental Association (ADA); six were from commercial organisations such as Colgate-Palmolive, Macleans and Oral B; and the majority were published by State/Territory Health Departments. The leaflets were appraised based on their textual framework, thoroughness, use of jargon terms and readability.

### Textual framework

Textual framework was assessed using three parameters:

• physical attributes;

• the use of relevant/instructional pictures; and

• the use of headings, subheadings and percentage of bulleted text.

The physical attributes of the leaflets were noted in terms of the format (e.g.: booklet, tri-fold brochure, or a flyer), and the number of pages.

The leaflets were given a score of “yes” for the use of relevant pictures if they followed the principle of dual code theory (visual and verbal elements in parallel) [29]. Examples included pictures of tooth brushing technique, illustration of the amount of toothpaste recommended for brushing children’s teeth, and pictures of cariogenic foods and drinks to avoid.

Each leaflet was examined for the use of headings, subheadings and the percentage of bulleted text using the Kool’s macro- and micro-coherence model of communication [30].

### Thoroughness/content

Each leaflet was appraised for the presence of information on topics within the scope of paediatric dentistry and the evidence base for the messages they delivered. These included: Early Childhood Caries and/or dental caries; diet; dental visits; tooth-brushing; fluoride; toothpaste use; non-nutritive behaviours; gum care; use of sipper cups; flossing; teething; trauma; tooth eruption and fissure sealants.

### Use of jargon text

Each leaflet was screened for the use of professional jargon. We identified a list of terms reported by other authors [28,31] as well as those present in the leaflets we studied.

### Readability analyses

The readability of each leaflet was calculated using three widely used indices in analysing health care materials: the Flesch-Kincaid grade level (FKGL), the Gunning Fog index (Fog) and the Simplified Measure of Gobbledygook (SMOG) [32]:

$$\text{FKGL}=\left(0.39\times \text{ASL}\right)+\left(11.8\times \text{ASW}\right)–15.59$$

$$\text{Fog}=0.4\left(\text{ASL}+\;\text{percentage}\;\text{of}\;\text{PSW}\right)$$

SMOG=3+√PSWcount

Where

ASL = average sentence length

ASW = average syllable per word

PSW = polysyllable word or word with more than 2 syllables

PSW count = number of PSW in a 30-sentence sample

All three formulae yield a numerical value that represents the grade level, or number of years of formal education required to comprehend the corresponding passage.

The content of all leaflets were entered into an automated online program to calculate their readabilities using the above formulae [33]. Abbreviations such as “e.g.” were edited to allow the automated program to perform the word and sentence counts correctly. The word and sentence count obtained by the online program were also confirmed manually.

## Results

### Textual framework

Table 1 provides an overview of the leaflets. The leaflets ranged from single page handouts, to tri-fold brochures, to eight page booklets. Out of the 40 leaflets, only four did not have relevant pictures, one from the ADA and three from New South Wales (NSW) Health. All leaflets used headings and sub-headings to display information and majority of them used 50 percent or more of bulleted text. Five used no bulleted text and 11 were entirely based on bulleted text.

**Table 1:** Summary of physical attributes of Australian paediatric oral health leaflets

**No.**	**Publisher**	**Source**	**Title**	**Format**	**Relevant picture**	**% Bulleted text**
1	ADA	Industry	7 tips for healthy baby teeth	2-page	No	100
2	ADA	I	Dental care for babies and young children	4-page booklet	Yes	25
3	Colgate	Commercial	Bright Smiles at Home	6-page trifold	Yes	75
4	Colgate	C	Oral health for infants and toddlers	8-page 4-fold	Yes	10
5	Colgate	C	Zero to six pre-school	6-page trifold	Yes	25
6	Colgate	C	oral health for children 3–12	8-page 4-fold	Yes	15
7	Macleans	C	Teaching your child good brushing habits	6-page trifold	Yes	10
8	Oral B	C	How do I care for my child’s teeth?	2-page single	Yes	80
9	Dental Health Services Victoria	Government	Tooth Tips for parents, grandparents & carers	2-page	Yes	100
10	Dental Health Services Victoria	G	Tooth Tips for parents, grandparents & carers	2-page	Yes	80
11	Dental Health Services Victoria	G	Stay Well fact sheet for parents	2-page	Yes	50
12	Dental Health Services Victoria	G	Play Well fact sheet for parents	2-page	Yes	40
13	Dental Health Services Victoria	G	Eat Well fact sheet for parents	2-page	Yes	30
14	Dental Health Services Victoria	G	Drink Well fact sheet for parents	2-page	Yes	10
15	Dental Health Services Victoria	G	Clean Well fact sheet for parents	2-page	Yes	20
16	Dental Health Services Victoria	G	How to brush your child’s teeth	1-page single	Yes	100
17	Northern Territory Government	G	Give your child’s teeth a healthy start	6-page trifold	Yes	0
18	Northern Territory Government	G	Cleaning your child’s teeth	1-page	Yes	0
19	Northern Territory Government	G	Do give you child, Don’t give your child	1-page	Yes	0
20	NSW Health	G	Healthy mouths for kids under 5	6-page trifold	Yes	100
21	NSW Health	G	Teach your baby to drink from a cup	6-page trifold	Yes	50
22	NSW Health	G	Eat Well Drink Well Clean Well Play Well Stay Well	6-page trifold	No	100
23	NSW Health	G	Caring for babies’ teeth	2-page single	No	50
24	NSW Health	G	Lift the Lip	3-page bifold	Yes	75
25	NSW Health	G	Tooth Smart	6-page trifold	No	100
26	NSW Health	G	Good Oral Health for Children	6-page trifold	Yes	100
27	NSW Health	G	Keeping smiling while you are pregnant	6-page trifold	Yes	100
28	NSW Health	G	Healthy Mouths for Aboriginal People	6-page trifold	Yes	100
29	Queensland Health	G	don’t rot your baby’s teeth	1-page single	Yes	75
30	Queensland Health	G	Looking after Young Mouth	13-page booklet	Yes	50
31	SA Health	G	Caring for your child’s smile	1-page single	Yes	100
32	SA Health	G	Give your child’s teeth a healthy start	6-page trifold	Yes	0
33	SA Health	G	DO give you child, Don’t give your child	1-page	Yes	0
34	WA Dental Health Services	G	Solid Kids have Healthy Teeth 0–2 Years Old	6-page trifold	Yes	75
35	WA Dental Health Services	G	Solid Kids have Healthy Teeth 2–5 Years Old	6-page trifold	Yes	75
36	WA Dental Health Services	G	Caring for your child’s smile (0–6 Years)	1- page	Yes	100
37	WA Dental Health Services	G	Thumbsucking and Dummies	4-page bifold	Yes	40
38	WA Dental Health Services	G	Teething	4-page bifold	Yes	30
39	WA Dental Health Services	G	Your Child’s First Dental Visit	4-page bifold	Yes	40
40	WA Dental Health Services	G	Brushing Toddler’s Teeth	4-page bifold	Yes	20

### Content analyses

Fourteen paediatric oral health topics were noted from the leaflets. These were: Early Childhood Caries/Dental Caries; Diet; Dental visits; Tooth brushing; Fluoride; Toothpaste amount; Non-nutritive behaviors; Gum care; use of Sipper cup; Flossing; Teething; Trauma; Tooth eruption; Fissure sealants.

Table 2 shows the coverage of topics by each leaflet. Four topics namely prevention of dental caries, diet, dental visits and tooth brushing were covered in over 75 percent of the leaflets. Dental caries prevention in particular was covered by over 90 percent of the leaflets. On the other hand, less than 20 percent of the leaflets had information on the use of fissure sealants, tooth development, trauma, flossing and teething. Only six leaflets covered 10 or more topics of interest.

**Table 2:** Thoroughness and content of Australian paediatric oral health leaflets*

**Leaflet number**	ECC**	**Diet**	**Visits**	**Brushing**	**Fluoride**	**Tooth-paste**	**Behaviours**	**Gum care**	**Sippy cups**	**Flossing**	**Teething**	**Trauma**	**Eruption**	**Sealants**
1	Y	Y	Y	Y	Y	-	Y	-	-	Y	-	-	-	-
2	Y	Y	Y	Y	Y	Y	Y	Y	Y	Y	Y	-	Y	Y
3	-	Y	Y	Y	Y	-	-	-	-	Y	-	-	-	Y
4	Y	Y	Y	Y	Y	Y	Y	-	-	-	Y	-	Y	-
5	Y	Y	Y	Y	Y	Y	Y	Y	-	-	Y	-	Y	-
6	Y	Y	Y	Y	Y	Y	Y	-	-	Y	-	Y	Y	Y
7	Y	Y	Y	Y	Y	Y	-	-	-	-	-	-	-	-
8	Y	Y	Y	Y	Y	Y	Y	Y	-	Y	-	Y	-	-
9	Y	Y	Y	Y	-	-	-	-	Y	-	Y	-	Y	-
10	Y	Y	Y	Y	Y	Y	Y	-	-	-	-	-	-	-
11	-	-	Y	-	-	-	Y	-	-	-	-	-	-	Y
12	-	-	Y	-	-	-	-	-	-	-	-	Y	-	-
13	Y	Y	Y	-	-	-	-	-	-	-	-	-	-	-
14	Y	Y	Y	-	Y	-	-	-	-	-	-	-	-	-
15	Y	-	Y	Y	Y	Y	-	-	-	-	-	-	-	-
16	Y	-	-	Y	-	Y	-	-	-	-	-	-	-	-
17	Y	Y	Y	Y	Y	-	Y	-	Y	-	-	-	-	-
18	-	-	-	Y	Y	-	-	-	-	-	-	-	-	-
19	Y	Y	-	-	-	-	-	-	-	-	-	-	-	-
20	Y	Y	-	Y	-	-	-	-	Y	-	-	-	-	-
21	Y	Y	-	-	-	-	-	-	Y	-	-	-	-	-
22	Y	Y	Y	Y	Y	Y	-	Y	Y	-	-	Y	-	Y
23	Y	Y	Y	Y	Y	Y	Y	-	-	-	-	-	-	-
24	Y	Y	Y	Y	Y	Y	-	Y	-	-	-	-	-	-
25	Y	Y	Y	Y	-	-	-	-	Y	-	-	-	-	-
26	Y	Y	Y	Y	Y	-	-	-	-	-	-	-	-	-
27	Y	Y	Y	Y	-	Y	-	-	-	Y	-	-	-	-
28	Y	Y	Y	Y	Y	Y	-	Y	-	Y	-	Y	-	-
29	Y	Y	Y	Y	Y	Y	Y	-	Y	-	-	-	-	-
30	Y	Y	Y	Y	Y	Y	Y	Y	-	-	Y	-	-	-
31	Y	Y	Y	Y	Y	Y	-	-	-	-	-	-	-	-
32	Y	Y	Y	Y	Y	-	Y	-	Y	-	-	-	-	-
33	Y	Y	-	-	-	-	-	-	-	-	-	-	-	-
34	Y	Y	Y	Y	Y	Y	Y	Y	Y	-	Y	Y	-	-
35	Y	Y	Y	Y	Y	Y	Y	Y	Y	Y	-	Y	-	-
36	Y	Y	Y	Y	Y	Y	Y	Y	Y	-	-	-	-	-
37	Y	Y	Y	-	-	-	Y	-	-	-	-	-	-	-
38	Y	-	Y	Y	Y	Y	-	Y	-	-	Y	-	Y	-
39	Y	-	Y	Y	Y	-	-	-	-	-	-	-	-	-
40	Y	-	-	Y	Y	Y	-	Y	-	-	-	-	-	-

*“Y” refers to a Yes.

**Early Childhood Caries.

Some leaflets stood out from others. One leaflet produced by the ADA: “Dental care for babies and young children”, provided the most comprehensive information, covering all topics except for dental trauma. Of all the commercial leaflets, one produced by Colgate-Palmolive: “Oral Health for children 3–12 years” covered 11 topics of interest. Amongst the government produced leaflets, two produced by Western Australia (WA) Health Department covered 11 topics. Two leaflets produced by Health Department of Northern Territory (NT), one by Health Department of Victoria (VIC), and one by Health Department of South Australia (SA) only covered two topics. Also, noteworthy NSW Health’s publication “Keep Smiling while you are pregnant”, one of the few paediatric oral health publications provided pre-natal oral health information for women.

The content analysis identified instances of incomplete or conflicting information. Specifically, most leaflets suggested parents to use a “pea-sized” amount of child fluoride toothpaste for cleaning their child’s teeth. However, two leaflets were generic and did not mention the amount of toothpaste to be used for cleaning their child’s teeth. Further, the recommended age of supervised toothbrushing varied across leaflets which was noted to be confusing. Most leaflets recommended that children should be supervised for brushing until they are eight years old. However, two leaflets mentioned the age of six and nine years, respectively.

### Use of jargon text

A list of 19 commonly used dental jargon terms were noted in the paediatric oral health leaflets. Of the list of jargon text, some commonly used terms were ECC, primary teeth, sealants and fluoride.

List of jargon terms section

Alignment

Antimicrobial

Appliance

Biofilm

Calcify

Dental caries

Disclosing tablets

Early childhood caries

Enamel

Fluoride

Gingivitis

Mouthguards

Nursing bottle caries

Orthodontics

Pits and fissures

Primary teeth

Sealants

Signs

Symptoms

### Readability analyses

**Figure 1:**
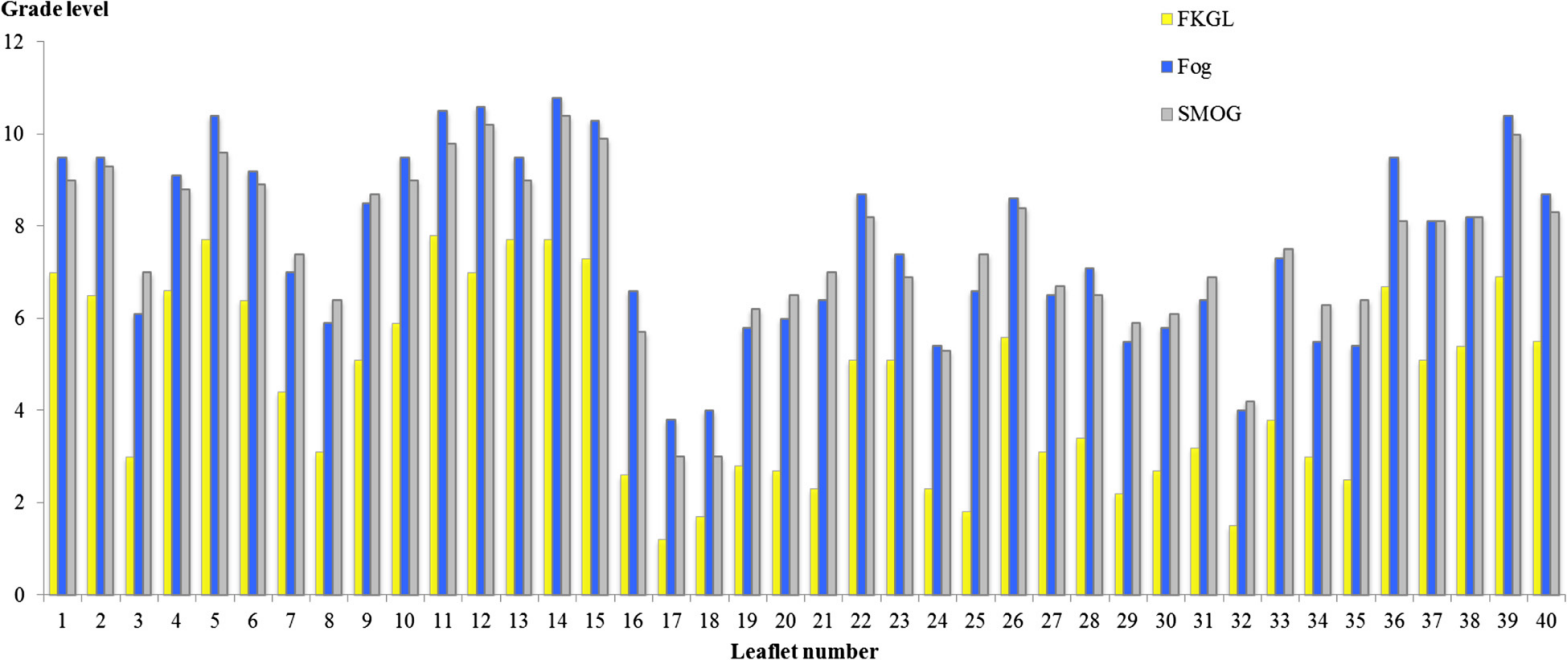
Readability indices scores of Australian paediatric leaflets.

Figure 1 shows the findings of readability indices for the leaflets. The lowest reading grade level was achieved by two leaflets entitled “Do give your child, Don’t give your child” and “Give your child’s teeth a healthy start” created by the NT and SA Health Departments, respectively. Their reading levels, based on Flesch-Kincaid, were noted at 1st grade (1.2 and 1.5, respectively) and both these publications use relevant pictures and easy-to-read text. Another easy-to-read leaflet was the NSW Health’s publication, “Tooth Smart”. This 6-page booklet registered at a 2nd grade reading level (FKGL score of 1.8) and was entirely based on bulleted text. Four leaflets, three by VIC Health Department and one by Colgate-Palmolive, yielded a FKGL score of 8. The most thorough leaflet that covered 13 out of 14 topics had a FKGL score of 6.5 but a Fog score of 9.5 and SMOG score of 9.3. The FKGL formula consistently yielded 2 to 3 grade levels below the Fog and SMOG formulae scores.

The average of the grade levels calculated by the FKGL, Fog and SMOG formulae were used to compare the readabilities of the leaflets. Among them, those produced by the commercial industry had a readability ranging from 5th to the 9th grade; those produced by the ADA had readability at the 8th or 9th grade; and those produced by State/Territory Health Departments had a readability ranging from the 3rd to the 9th grade. The average readability of leaflets from ADA was 8th grade (Mean-8.4, SD-0.1); commercial industry was 7th grade (Mean-7.1, SD -1.7); and those from the State/Territory Health Departments was 6th grade (Mean-6.3, SD-1.9).

## Discussion

Educating parents on child oral health related issues is one of the most important steps during the primary socialisation process. Leaflets form an important link in the chain of communication between oral health professionals, the parents and the child. As noted in this study, although many paediatric oral health leaflets exist in Australia, they vary in content and readability. It was noted that leaflets that were adequate in terms of low literacy demand often had minimal information on child oral health. Conversely, the leaflets that had comprehensive information required higher literacy skills, which may be difficult for parents from disadvantaged backgrounds or those from linguistically diverse communities. Our results suggest that is likely that leaflets with simple messages and low literacy demand are read by more people compared to comprehensive leaflets.

The present study utilised three measures of readability assessment (FKGL, Fog, and SMOG) due to their simplicity and widespread use [32]. The grade levels calculated by FKGL were lower compared to Fog and SMOG, while the difference between Fog and SMOG were small (Figure 1). It is pertinent to note that these formulae are validated against the McCall- Crabs Passages [32]. These differences are noted because the FKGL predicts the grade level based on 75 percent comprehension, while the Fog and SMOG predict the grade level based on 90 percent and 100 percent comprehension respectively. Although other methods to assess the readability of education materials such as the SAM method (Suitability Assessment of Materials) are available [27], these were deemed inappropriate for this study as some leaflets contained less than 100 words.

The use of formula based readability assessment provided useful information on only one aspect of readability i.e. the level of education required to comprehend the leaflet. Other aspects such as font size, font colour, the use of bold and italics text, use of bulleted text; use of instructional pictures; use of simplified sentences, and sentence length which contribute to the overall readability [28,34,35] are not assessed by the readability indices. However, in this study, some of these aspects such as the use of bold and bullet text and the use of pictures were assessed. It has also been noted elsewhere that readability formulae should only be used as a guide to assess the reading difficulty of a text as they do not take into account other factors that can influence comprehension [36] such as the use of active and passive verbs, the way the information is organised on a page and the reader’s motivation and level of prior knowledge [37]. Blinkhorn and Verity [38] noted that dental professionals use professional terminology that may be incomprehensible to a common person and that readability formulae may therefore underestimate the difficulty of a text.

The content analyses of Australian leaflets revealed the prevalence of conflicting health education messages as noted by other researchers [31]. It is noteworthy that research in experimental psychology and marketing highlights that humans have a cognitive preference for picture-based, rather than text-based information: the so-called picture superiority effect [39,40]. Although majority of the leaflets had relevant pictures, it was surprising to note that some leaflets did not have photographs to convey important messages to parents of young children such as how to brush the child’s teeth and the amount of toothpaste to be used. Similar to other studies [27,28,31], the present study identified aspects of oral hygiene instruction such as how to brush teeth that were not covered by majority of the leaflets. Pictures have been a useful tool in health sciences to covey health messages and would be useful for Australian dental professionals to convey the correct information about toothpaste use and other aspects of oral hygiene using illustrations as it is reported that patients retain more health information to visual presentations [40,41].

Although there is no gold standard tool to assess a patient’s oral health literacy at this stage, several instruments are being developed as research in this aspect of oral health is increasing relatively [21]. It is now noted that oral health literacy is an important link between health behaviours and oral health outcomes [20]. Although there is evidence that improving patients’ literacy can improve health outcomes [42], it is still unclear if improving patient education materials can lead to better health outcomes [43]. However, it is pertinent to note that producers of dental health education materials should be aware of several parameters when designing leaflets. These include general readability of leaflets; coverage of important health topics with simple and consistent messages; the use of pictures to convey health messages; and avoiding the use of jargon terms. Health education leaflets that are clear, concise, consistent and thorough are a simple way to bridge the communication gap between the oral health professional, parent and the child.

The present study has several limitations; although several criteria were used to evaluate the readability of the leaflets, there were some aspects of the general readability that were not assessed. These include the use of bright colours, use of active and passive verbs, the use of italics to emphasize information, and the advantageous use of white space [34]. Second, the list of dental jargon terms noted in this study were subjective. However, it is important to note that other authors have noted similar words in their studies [28,31]. Thirdly, although all possible Australian sources were searched to collect leaflets, it may be possible that some paediatric oral health leaflets were inadvertently missed. Finally, the current evaluation does not include the opinion of the parents which will be prudent to re-design of the leaflets in future.

## Conclusions

Australian paediatric oral health education materials are readily available, yet their quality and readability vary widely. Leaflets produced by local Health Departments are more readable compared to commercial and industry counterparts. The results show that a large number of paediatric dental leaflets may be difficult to read for disadvantaged populations in Australia. A redesign of these leaflets while taking literacy into consideration is suggested.

## Competing interests

The authors declare that they have no competing interests.

## Authors’ contributions

AA conceptualised the study. AA, ZK, ASFL collected all the leaflets and conducted the content analysis. LD and MH was consulted in case of any discrepancy. All authors wrote the draft version of the manuscript and approved the final version.

## Pre-publication history

The pre-publication history for this paper can be accessed here:

http://www.biomedcentral.com/1472-6831/14/111/prepub
